# Glutathione S-transferase M1, T1, and P1 polymorphisms and periodontitis in a Caucasian population: a case-control study

**DOI:** 10.1186/s12903-024-04034-x

**Published:** 2024-02-28

**Authors:** Pavla Izakovicova, Antonin Fassmann, Ladislav Dusek, Lydie Izakovicova Holla

**Affiliations:** 1 Faculty of Medicine, Masaryk University, Kamenice 753/5, Brno, 625 00 Czech Republic; 2 Clinic of Stomatology, Institution Shared with St. Anne’s University Hospital, Faculty of Medicine, Masaryk University, Pekarska 664/53, Brno, 602 00 Czech Republic; 3 Institute of Biostatistics and Analyses, Faculty of Medicine, Masaryk University, Kamenice 753/5, Brno, 625 00 Czech Republic; 4 Department of Pathophysiology, Faculty of Medicine, Masaryk University, Kamenice 753/5, Brno, 625 00 Czech Republic

**Keywords:** Gene, Glutathione S-transferase, GSTs, Periodontitis, Polymorphism

## Abstract

**Background:**

Glutathione S-transferases (GSTs) play important roles in protecting cells against oxidative stress and toxic chemicals. This study aimed to investigate the distribution of *GSTM1*, *GSTT1*, and *GSTP1* variants and their roles in periodontitis susceptibility in a Caucasian population.

**Methods:**

We analyzed 406 participants, including 204 healthy controls and 203 periodontitis patients. A multiplex polymerase chain reaction (PCR) approach was used to analyze *GSTM1* and *GSTT1* loci. *GSTP1* variants were detected by PCR-haplotyping method in a subgroup of participants (N = 350). Chi-square or Fisher´s exact tests were used to compare genotypic and allelic differences. The Bonferroni method was applied to correct for multiple comparisons (p_corr_).

**Results:**

The *GSTM1* genotype distribution did not differ significantly between controls and periodontitis patients (p = 0.44). Additionally, the wild/null genotypes of *GSTT1*, Ile105Val and Ala114Val frequencies of *GSTP1* were not significantly different between the two groups after correction for multiple comparisons (p = 0.05, p = 0.55, p = 0.02, p_corr_>0.05, respectively). The *GSTM1* and *GSTP1* Ile105Val gene variants were similarly distributed between non-smokers and smokers in both groups (p = 0.38, p = 0.20, and p = 0.14, p = 0.35, respectively). However, the wild genotype of the *GSTT1* and Ala114Ala variant of the *GSTP1* genes were present more frequently in non-smoking periodontitis patients than in non-smoking controls (p = 0.03, p_corr_>0.05, and p = 0.009, p_corr_>0.05, respectively) although their frequencies did not differ between smoking periodontitis patients and smoking controls (p = 0.23, p = 0.68, respectively).

**Conclusions:**

This study in a Czech Caucasian population did not confirm a highly significant association between *GST* gene variants and susceptibility to periodontitis, as previously reported by Arshad and colleagues in Pakistanis. However, a weak relationship between *GSTT1* and *GSTP1* rs1138272 polymorphisms and periodontitis in non-smokers was observed.

## Background

Periodontal disease affects up to 90% of the population, making it one of the most common oral problems [1]. Periodontitis is a complex infectious disease attributed to the presence of bacteria in dental plaque and is characterized by chronic inflammation and destruction of the supporting structures of teeth. Although dental plaque bacteria are necessary for disease development, the immune response of the body is essential. The host immune responses that contribute to periodontitis are also associated with oxidative stress [2]. Antioxidant mechanisms act against reactive oxygen species (ROS) before they cause oxidative damage to tissues and cells [3]. Additionally, smoking is a strong risk factor for periodontitis, and tobacco smoke can release several substances capable of inhibiting cellular immune responses. For detoxification, conjugation with glutathione phase II enzymes, such as glutathione-S-transferases (GSTs) is necessary [4].

The primary role of glutathione-S-transferases (GSTs) is to detoxify reactive electrophilic compounds, including environmental toxins and oxidative stress products. The GSTs include seven classes: alpha (*α*, GSTA), mu (*µ*, GSTM), pi (*π*, GSTP), sigma (*σ*, GSTS), theta (*θ*, GSTT), omega (*ω*, GSTO), and zeta (*ξ*, GSTZ) [5]. The *GSTM1* gene is located on chromosome 1 (1p13.3) and the *GSTT1* gene is located on chromosome 22 (22p11.23). The most common mutation in these two genes is the whole null genotype, which results in a lack of enzymatic activity and may cause upregulation of oxidative stress [6]. The human *GSTP1* gene is located on chromosome 11 (11q13.2) and contains two common polymorphisms in exon 5, namely, rs1695 (A313G, Ile105Val), and in exon 6, namely, rs1138272 (C341T, Ala114Val), with possible functional effects. Consequently, the changes of Ile or Ala to Val, which are in the electrophile-binding active site of the GSTP peptide, may result in subtle alterations in the a-helical and/or superhelical structure, which can affect H-site architecture and ultimately result in differences in substrate binding affinities and catalytic activities between the GSTP enzymes [7].

A recent case-control study in a Pakistani population reported that the absence of *GSTM1*, the presence of *GSTT1*, and the mutant allele (G) at rs1695 in the *GSTP1* gene may be associated with susceptibility to periodontitis [8]. This study aimed to determine the frequencies of *GSTM1*, *T1*, and *P1* variants and analyze their role in periodontitis susceptibility in a Caucasian (Czech) population of non-smokers and smokers.

## Methods

### Study design, clinical examination, and sample collection

This case-control study follows the principles of the Declaration of Helsinki. This study was approved by the Ethics Committee of Masaryk University, Faculty of Medicine (No. 15/2009, No. 13/2013) and St. Anne’s Faculty Hospital Brno (without No. /2005). All participants provided written informed consent prior to inclusion in the study. All DNA samples were obtained from the biobank of the Clinic of Stomatology, St. Anne´s Faculty Hospital and Faculty of Medicine, and Department of Pathophysiology, Faculty of Medicine, from participants recruited from 2005 to 2015.

The participants with periodontitis (N = 203) were patients at the Clinic of Stomatology. All participants had at least 20 remaining teeth and were in good general health. Their Community Periodontal Index of Treatment Needs (CPITN) [9] was 3 or 4. Periodontitis was diagnosed based on clinical examination and radiographic evaluation. Probing depth (PD) and clinical attachment loss (CAL) were determined using a UNC-15 periodontal probe (HuFriedy, Chicago, IL, USA) from six sites on each tooth. The gingival health status was determined using the modified Löe-Silness gingival index (GI) [10] as described previously [11], and alveolar bone loss was determined radiographically and assessed using the Mühlemann index [12]. All periodontitis patients fulfilled the diagnostic criteria for chronic periodontitis (CP) defined according to CAL levels by the International Workshop for the Classification of Periodontal Diseases and Conditions for Chronic Periodontitis [13]. The inclusion criteria for patients with generalized CP were as follows: ≥ 30% of the teeth were affected, PD of ≥ 4 mm, and the amount of CAL was consistent with the presence of mineralized plaque. All our patients would have belonged to Stages 2–4 and most of them to Grade B according to the new (currently valid) classification [14].

Controls (N = 204) were recruited from patients who were referred to the Clinic of Stomatology for various reasons (e.g., preventive dental examinations, dental caries, and orthodontic consultations) during the same period as the periodontitis patients. They were of similar age, sex, smoking status, and had no previous diagnosis of periodontitis. Moreover, they had at least 20 remaining teeth, were in good general health, and with a CPITN of < 3.

The exclusion criteria were the presence of systemic diseases such as diabetes mellitus, cardiovascular disorders, pregnancy or lactation, malignant diseases; use of anti-inflammatory drugs or antibiotics for at least six weeks before the recruitment period; and an inability to provide consent, as described previously [15].

The participants were classified into the groups of non-smokers (participants who never smoked) and smokers (former smokers for ≥ 5 pack-years or current smokers) [16]. Pack-years were calculated by multiplying the number of cigarettes smoked per day by the number of years that a person smoked.

### Isolation of genomic DNA

Genomic DNA was isolated from peripheral blood leukocytes by standard proteinase K digestion and phenol-chloroform extraction method [17].

### Genetic analysis

#### Analysis of GSTM1 and GSTT1 gene variants

The analysis of *GSTM1* and *GSTT1* genes was simultaneously performed in a single assay using a slightly modified multiplex polymerase chain reaction (PCR) method [18] with primers listed in Table 1 and the details described previously [19]. PCR was performed using a SensoQuest Labcycler (Schoeller, Germany). The presence or absence of *GSTM1* and *GSTT1* genes was detected as a band at 215 bp (corresponding to *GSTM1*), 480 bp (corresponding to *GSTT1*), and 312 bp (corresponding to *CYP1A1* as an internal control).

**Table 1:** Multiplex PCR primers for *GSPM1* and *GSTT1* polymorphisms

Gene	Sense primer	Antisense primer	Product size (bp)
*GSTM1*	5´-gAACTCCCTgAAAAgCTAAAgC-3´	5´-gTTgggCTCAAATATACggTg-3´	215
*GSTT1*	5´-TTCCTTACTggTCCTCACATCTC-3´	5´-TCACCggATCATggCCAgCA-3´	480
Primers for *CYP1A1* exon 7 (internal control)	5´-gAACTgCCACTTCAgCTgTCT-3´	5´-CAgCTgCATTTggAAgTgCTC-3´	312

#### Analysis of GSTP1 genetic polymorphisms

To detect two *GSTP1* polymorphisms (Ile105Val and Ala114Val) simultaneously, an assay with forward and reverse allele-specific primers enabling the identification of *cis/trans* orientation (PCR haplotyping) [20], with slight modifications, was applied. The primers and product sizes are listed in Table 2. All reaction mixtures contained control primers for human growth hormone as described previously [19].

**Table 2:** Polymerase chain reaction – sequence specific primers (PCR-SSP) for *GSPP1* polymorphisms

Alleles	Sense primer	Antisense primer	Product size (bp)
*GSTP1* 105Ile-114Ala (GSTP1_A)	5´-ggACCTCCgCTgCAAATACA-3´	5´-CACATAgTCATCCTTgCCCg-3´	928
*GSTP1* 105Val-114Ala (GSTP1_B)	5´-ggACCTCCgCTgCAAATACg-3´	5´-CACATAgTCATCCTTgCCCg-3´	928
*GSTP1* 105Val-114Val (GSTP1_C)	5´-ggACCTCCgCTgCAAATACg-3´	5´-CACATAgTCATCCTTgCCCA-3´	928
*GSTP1* 105Ile-114Val (GSTP1_D)	5´-ggACCTCCgCTgCAAATACA-3´	5´-CACATAgTCATCCTTgCCCA-3´	928
Primers for human growth hormone (internal control)	5´-gCCTTCCCAACCATTCCCTT-3´	5´-TCACggATTTCTgTTgTgTTTC-3´	426

### Statistical analysis

The power analysis for Fisher’s exact test was computed with the following settings: assumed sample size for the control/treatment group N = 200/200, power 80%, and level of statistical significance a = 0.05 [21, 22]. The chi-square test was used to assess Hardy-Weinberg equilibrium (HWE) and to compare genotype differences. Allele frequencies were calculated from the observed genotypes using Fisher’s exact test. The Bonferroni method was employed to eliminate the issue of multiple hypothesis testing (p_corr_). One-way analysis of variance (ANOVA) and Kruskal-Wallis ANOVA were used to compare continuous variables between independent groups. All statistical analyses were performed using Statistica version 14 (StatSoft Inc., Tulsa, Okla., USA).

## Results

Power calculations were performed for a wide range of endpoint relative frequencies in the control and treatment cohorts (0.25–0.75). Given the settings, the planned sample size allowed the detection of differences in the occurrence of examined markers ± 11% as statistically significant, which was confirmed by the estimation of 95% confidence limits of odds ratio (OR).

The characteristics of 407 participants comprising 204 healthy controls (108/96 males/females; 48.6 ± 6.0 years, mean age ± standard deviation, SD) and 203 periodontitis patients (99/104 males/females; 54.3 ± 7.4 years) are listed in Table 3. Smoking status was known in 187 (91.7%) healthy controls and 187 (92.1%) periodontitis patients. The groups did not differ in the male/female ratio (p > 0.05) or smoking status (p > 0.05); however, the periodontitis patients were older than the healthy individuals (p < 0.05). Periodontal health parameters, such as PD, CAL, GI, and Mühlemann index, were significantly different between healthy participants and periodontitis patients (p < 0.001; Table 3).

**Table 3:** Demographic data and clinical characteristics of controls and periodontitis patients

	Controls (N = 204)	Periodontitis patients (N = 203)	p level
Age (years, mean ± SD)	48.6 ± 6.0	54.3 ± 7.4	< 0.05
Sex: Female N (%)	96 (47.1)	104 (51.2)	
Male N (%)	108 (52.9)	99 (48.8)	0.229†
Smokers N (%)	58 (31.0)	57 (30.5)	0.506†
Probing pocket depth (mm) (mean ± SD)	1.51 ± 0.27 1.36 ± 0.24 (non-smokers) 1.63 ± 0.30 (smokers)	5.26 ± 0.71 5.39 ± 1.0 (non-smokers) 5.76 ± 0.95 (smokers)	< 0.001*
Clinical attachment loss (mm) (mean ± SD)	0.00 ± 0.00	4.24 ± 1.35 4.17 ± 1.34 (non-smokers) 4.69 ± 1.36 (smokers)	< 0.001*
Gingival index (mean ± SD)	0.40 ± 0.31 0.42 ± 0.28 (non-smokers) 0.39 ± 0.35 (smokers)	0.89 ± 0.35 0.95 ± 0.40 (non-smokers) 0.91 ± 0.23 (smokers)	< 0.001*
Mühlemann index♦ (mean ± SD)	0.00 ± 0.00	2.08 ± 0.66 2.03 ± 0.66 (non-smokers) 2.18 ± 0.66 (smokers)	< 0.001*

†Analyzed with Fisher´s exact test, the remaining values were analyzed with the t-test

♦This index measures decreases in the alveolar bone. The mean value of the score in periodontitis patients was 2.08 (± 0.66), i.e. a loss of the alveolar bone from 25–50% of the root

*Statistically significant p_corr_ < 0.05

Table 4 shows the distribution of *GST* variants in healthy controls and periodontitis patients. The genotype frequencies in healthy controls were in accordance with HWE (p > 0.05, data not shown). We found no significant differences in the *GSTM1*, *GSTT1*, or *GSTP1* genotype frequencies between the two groups (p_corr_ > 0.05).

**Table 4:** GSTs genotype frequencies in periodontitis patients and controls

Genotypes	Controls (N = 204)	Periodontitis patients (N = 203)	p level	OR (95%CI)
*GSTM1*				
Wild	100 (49.0)	97 (47.8)	0.44	1.00
Null	104 (51.0)	106 (52.2)		1.05 (0.71–1.55)
*GSTT1*				
Wild	152 (74.5)	166 (81.8)	0.05*	1.00
Null	52 (25.5)	37 (18.2)		0.65 (0.40–1.05)
*GSTM1/GSTT1*				
Both wild	71 (34.8)	78 (38.4)	0.61	1.00
Either null	110 (53.9)	107 (52.7)		0.89 (0.58–1.34)
Both null	23 (11.3)	18 (8.9)		0.71 (0.36–1.43)
*GSTP1* Ile105Val (rs1695, A313G)#				
AA (Ile/Ile)	75 (50.0)	108 (54.0)	0.55	1.00
AG (Ile/Val)	66 (44.0)	77 (38.5)		0.81 (0.52–1.26)
GG (Val/Val)	9 (6.0)	15 (7.5)		1.16 (0.48–2.78)
*GSTP1* Ala114Val (rs1138272, C341T) #				
CC (Ala/Ala)	126 (84.0)	178 (89.0)	0.02*	1.00
CT (Ala/Val)	24 (16.0)	17 (8.5)		0.50 (0.26–0.97)
TT (Val/Val)	0 (0.0)	5 (2.5)		NA
*GSTP1*#				
*A/*A	75 (36.8)	108 (53.2)	0.10	1.00
*A/*B	47 (23.0)	62 (30.5)		1.09 (0.68–1.76)
*A/*C	19 (9.3)	15 (7.4)		0.55 (0.26–1.15)
*B/*B	4 (2.0)	8 (3.9)		1.39 (0.40–4.78)
*B/*C	5 (2.4)	2 (1.0)		0.28 (0.05–1.47)
*C/*C	0 (0.0)	5 (2.5)		NA

#*GSTP1* gene polymorphisms were analysed in 150 healthy controls and 200 periodontitis patients only

*p_corr_ > 0.05

Table 5 shows the GSTs frequencies in healthy controls and periodontitis patients according to their smoking status. The *GSTM1* gene variants were similarly distributed between non-smokers and smokers. However, the wild genotype of the *GSTT1* and Ala114Ala variant of the *GSTP1* genes were present more frequently in the non-smoking periodontitis patients than in non-smoking controls (p = 0.03, p_corr _> 0.05; p < 0.009, p_corr _> 0.05, respectively). Conversely, the *GST* variants did not differ significantly between smoking periodontitis patients and smoking controls (p > 0.05).

**Table 5:** *GSTs* genotype frequencies in periodontitis patients and controls according to their smoking status^♦^

Genotypes	Non-smoking controls (N = 129)	Non-smoking periodontitis patients (N = 130)	p level	OR (95%CI)	Smoking controls (N = 58)	Smoking periodontitis patients (N = 57)	p level	OR (95%CI)
*GSTM1*								
Wild	59 (45.7)	63 (48.5)	0.38	1.00	31 (53.4)	25 (43.9)	0.20	1.00
Null	70 (54.3)	67 (51.5)		0.90 (0.55–1.46)	27 (46.6)	32 (56.1)		1.47 (0.70–3.06)
*GSTT1*								
Wild	96 (74.4)	110 (84.6)	0.03*	1.00	48 (82.8)	43 (75.4)	0.23	1.00
Null	33 (25.6)	20 (15.4)		0.53 (0.28–0.98)	10 (17.2)	14 (24.6)		1.56 (0.63–3.88)
*GSTM1/GSTT1*								
Both wild	44 (34.1)	50 (38.5)	0.07	1.00	24 (41.4)	21 (36.8)	0.11	1.00
Either null	67 (51.9)	73 (56.2)		1.04 (0.62–1.76)	31 (53.4)	26 (45.6)		0.96 (0.44–2.10)
Both null	18 (14.0)	7 (5.4)		0.34 (0.13–0.90)	3 (5.2)	10 (17.5)		3.81 (0.92–15.71)
*GSTP1* Ile105Val (rs1695 A313G)								
AA (Ile/Ile)	38 (43.7)	72 (55.8)	0.14	1.00	32 (66.7)	30 (53.6)	0.35	1.00
AG (Ile/Val)	42 (48.3)	45 (34.9)		0.57 (0.32–1.01)	14 (29.2)	24 (42.9)		1.83 (0.80–4.18)
GG (Val/Val)	7 (8.0)	12 (9.3)		1.11 (0.40–3.04)	2 (4.2)	2 (3.6)		0.94 (0.12–7.08)
*GSTP1* Ala114Val (rs1138272 C341T)								
CC (Ala/Ala)	69 (79.3)	113 (87.6)	0.009*	1.00	44 (91.7)	50 (89.3)	0.68	1.00
CT (Ala/Val)	18 (20.7)	11 (8.5)		0.37 (0.17–0.84)	4 (8.3)	6 (10.7)		1.32 (0.35–4.98)
TT (Val/Val)	0 (0.0)	5 (3.9)		NA	0 (0.0)	0 (0.0)		NA
*GSTP1*								
*A/*A	38 (43.7)	72 (55.8)	0.04*	1.00	32 (66.7)	30 (53.6)	0.54	1.00
*A/*B	29 (33.3)	36 (27.9)		0.66 (0.35–1.23)	10 (20.8)	18 (32.1)		1.92 (0.77-4:82)
*A/*C	13 (14.9)	9 (7.0)		0.37 (0.14–0.93)	4 (8.3)	6 (10.7)		1.60 (0.41–6.23)
*B/*B	2 (2.3)	5 (3.9)		1.32 (0.24–7.12)	2 (4.2)	2 (3.6)		0.94 (0.12–7.08)
*B/*C	5 (5.7)	2 (1.6)		0.21 (0.04–1.14)	0 (0.0)	0 (0.0)		NA
*C/*C	0 (0.0)	5 (3.9)		NA	0 (0.0)	0 (0.0)		NA

^♦^Smoking status was known only in subgroups of periodontitis patients and controls

*p_corr_ > 0.05

## Discussion

Periodontitis is a chronic and extremely widespread disease, the multifactorial etiology of which is affected by complex environmental, behavioral, and genetic factors [23]. Recently, the genetic variability of *GSTs* has been reported as a susceptibility factor for periodontitis in a Pakistani population. In an investigated set of 201 controls and 203 periodontitis patients, the researchers detected that the absence of *GSTM1*, presence of *GSTT1*, and mutant allele (G) at rs1695 in the *GSTP1* gene might be associated to susceptibility to periodontitis [8]. However, this is not consistent with the results of this study, which involved a similar number of participants (204 healthy controls and 203 periodontitis patients) but found no significant differences in the distribution of *GSTM1, GSTT1*, and *GSTP1* gene variants after correction for multiple comparisons between the two groups. Furthermore, as harmful products derived from cigarette smoke reportedly affect the association between several genes and periodontal status [24–26], we also explored the interaction between *GST* polymorphisms and smoking in the development of periodontitis. In our study, the *GSTM1* gene variants were similarly distributed between non-smokers and smokers. However, the wild genotype of *GSTT1* and Ala114Ala variant of the *GSTP1* genes were present more frequently in non-smoking periodontitis patients than in non-smoking controls.

GSTs are involved in the neutralization of hydroperoxides derived from lipoperoxidation processes during oxidative stress caused by periodontal inflammatory processes and in the detoxification of xenobiotics, especially tobacco-derived substances, such as polycyclic aromatic hydrocarbons and hydroxylated metabolites of benzo-α-pyrene [27–29]. Kim et al. [30] first described a significantly increased risk of periodontal disease among participants (115 with chronic periodontitis and 126 controls) with *GSTM1*(+) genotype (OR = 2.1, 95%CI = 1.3–3.6) in a Korean population. Those with the *GSTM1*(+) genotype had a significant increase in periodontitis risk among smokers (OR = 3.1, 95%CI = 1.5–6.6) and a moderate risk increase among non-smokers (OR = 1.8, 95%CI = 1.0-3.1). Conversely, Concolino et al. [4] showed a significant association between both aggressive (N = 14) and chronic (N = 69) forms of periodontitis and the *GSTM1*-null variant in Italians (OR = 3.59, 95%CI = 1.66–7.84) regardless of smoking, patients’ age, sex, and hygienic habits. However, no significant differences were found in *GSTT1* genotypes. In addition, in their pilot study, Ortega et al. [31] analyzed *GSTM1*, *GSTT1*, and *GSTP1* polymorphisms in 60 Mexicans with chronic periodontitis (30 non-smokers and 30 smokers). Polymorphisms in the *GSTT1* and *GSTP1* genes were not significantly different between smokers and non-smokers; however, the *GSTM1*(+) genotype was significantly more frequent in smokers with periodontitis (p ≤ 0.05). However, compared to historical data from a healthy Mexican population, periodontitis patients showed a higher frequency of null and mutant polymorphisms in *GSTM1*, *T1*, and *P1* [31–33]. Finally, a recent study by Saravanan et al. in 100 participants from a South Indian population did not reveal any significant association between *GSTP1* (rs1695) polymorphism and periodontitis [34]. However, the *GSTM1* and *GSTT1* null genotypes have been found to be associated with an increased risk of apical periodontitis [35] and a significantly enhanced risk of developing oral cancer [36].

The inconsistent conclusions reported in various studies could be attributed to the different ethnic backgrounds, as the frequency of *GSTs* polymorphic alleles between populations can vary significantly; different inclusion and exclusion criteria; or different methodological approaches to the analyses [37].

Certain limitations of this study should be considered. The main drawback is that the number of smokers is relatively small. This may lead to potential biases that could influence the study findings. Moreover, case-control study designs tend to produce false-positive results when cases and controls are from different population strata. However, in this study, the participants were selected from a small region of South Moravia in the Czech Republic, and thus the population studied was homogeneous. Furthermore, the participants in this study were all Caucasians; therefore, the effect of ethnicity could not be assessed. Finally, the association between gene polymorphisms and serum GST levels was not examined in this study, although it has been reported previously [38].

## Conclusion and clinical relevance

This study did not find a highly significant association between *GST* gene variants and susceptibility to periodontitis in a Caucasian population, as previously reported for Pakistanis. Weak relationships between *GSTT1* and *GSTP1* rs1138272 polymorphisms and periodontitis in non-smokers were observed in this study. From a clinical perspective, the analyzed *GST* polymorphisms cannot be used as markers of an increased risk of developing periodontitis in the Caucasian population, in contrast to the Asian population, where the presence of *GSTT1* and a mutant allele (G) at rs1695 in the *GSTP1* gene may be considered risk factors for genetic susceptibility to these disorders. The validity of these results must be confirmed in studies with robust sample sizes and independent cohorts of different ethnic origins.

## Data Availability

The datasets used and/or analyzed in the current study are available from the corresponding author upon reasonable request.

## Abbreviations

Ala: Alanine
ANOVA: Analysis of variance
bp: base pair
CAL: Clinical attachment loss
CI: Confidence interval
CP: Chronic periodontitis
CPITN: Community Periodontal Index of Treatment Needs
CYP1A1: Cytochrome P450 A1
DNA: Deoxyribonucleotide acid
GI: Gingival index
GSTs: Glutathione S-transferases
GSTM1: Glutathione-S-transferase µ-1
GSTP1: Glutathione-S-transferase *π* -1
GSTT1: Glutathione-S-transferase *θ* -1
HWE: Hardy-Weinberg equilibrium
Ile: Isoleucine
LD: Linkage disequilibrium
N: Number of individuals
NA: Non-applicable
OR: Odds ratio
PCR: Polymerase chain reaction
PCR-SSCP: Polymerase chain reaction – sequence-specific primers
PD: Probing depth
ROS: Reactive oxygen species
SD: Standard deviation
Val: Valine
